# Essential Oils and Their Constituents Targeting the GABAergic System and Sodium Channels as Treatment of Neurological Diseases

**DOI:** 10.3390/molecules23051061

**Published:** 2018-05-02

**Authors:** Ze-Jun Wang, Thomas Heinbockel

**Affiliations:** Department of Anatomy, Howard University College of Medicine, 520 W Str., NW, Washington, DC 20059, USA

**Keywords:** essential oils, terpenes, GABA receptor, sodium channel, transient receptor potential (TRP) channel, pain, epilepsy, analgesics, anticonvulsant, anxiolytic, antinociception, CNS, sensory neurons

## Abstract

Essential oils and the constituents in them exhibit different pharmacological activities, such as antinociceptive, anxiolytic-like, and anticonvulsant effects. They are widely applied as a complementary therapy for people with anxiety, insomnia, convulsion, pain, and cognitive deficit symptoms through inhalation, oral administration, and aromatherapy. Recent studies show that essential oils are emerging as a promising source for modulation of the GABAergic system and sodium ion channels. This review summarizes the recent findings regarding the pharmacological properties of essential oils and compounds from the oils and the mechanisms underlying their effects. Specifically, the review focuses on the essential oils and their constituents targeting the GABAergic system and sodium channels, and their antinociceptive, anxiolytic, and anticonvulsant properties. Some constituents target transient receptor potential (TRP) channels to exert analgesic effects. Some components could interact with multiple therapeutic target proteins, for example, inhibit the function of sodium channels and, at the same time, activate GABA_A_ receptors. The review concentrates on perspective compounds that could be better candidates for new drug development in the control of pain and anxiety syndromes.

## 1. Introduction

Essential oils (EOs) are concentrated hydrophobic liquid containing volatile aroma compounds which are extracted from herbs, flowers, and other plant parts. Oil is “essential” in the sense that it contains the “essence of” the plant’s fragrance. They are recommended for or encouraged to be applied as a complementary therapy for people with anxiety, pain, bipolar disorder, attention deficit hyperactivity disorder, and depression [1,2]. EOs can be absorbed into the body by oral administration, inhalation, diffusers, baths, and massages. Many studies show that EOs were effective in reducing pain, anxiety, and stress symptoms in animal models and humans with different CNS disorders [1,2]. EO constituents belong mainly to two chemical groups: terpenoids (monoterpenes and sesquiterpenes) and some phenylpropanoid derivatives. Terpenoid group compounds are usually fairly hydrophobic with molecular weights below 300 Daltons [3].

Activation of the γ-aminobutyric acid (GABA) receptor system and the blockade of neuronal voltage-gated sodium channels (Na^+^ channels) are essential for the overall balance between neuronal excitation and inhibition which is vital for normal brain function and critical for the central nervous system (CNS) disorders. It has been suggested that EO constituents could exert their biological activities through modulating the GABAergic system and inhibiting Na^+^ channels [4,5]. GABA is the major inhibitory neurotransmitter in the CNS and the GABA receptor system exerts a major inhibitory function in the brain. The dysfunction or deficiency of the GABAergic system has been implicated in epilepsy, pain, and anxiety [6]. Neuronal voltage-gated Na^+^ channels mediate the propagation of action potentials along axons, and thus, are thought to be important targets of antiseizure drugs. Local anesthetics and analgesics prevent the transmission of nerve impulses via their binding to Na^+^ channels. Two main types of Na^+^ currents, termed tetrodotoxin (TTX)-sensitive and TTX-resistant, have been identified in the dorsal root ganglion [7,8]. Studies on Na^+^ channels have demonstrated a greater involvement of Nav1.7, a predominant subtype of TTX-sensitive sodium channels expressed principally in peripheral neurons [8], in inflammatory pain [9,10] and in pain sensation [11,12]. 

Recently, many studies have addressed the potential of natural EOs for treatment of anxiety, convulsion, and pain in humans and in rodents or fish neuropathic models, and the mechanisms underlying the pharmacological profile. The main constituents of EOs were isolated and chemically elucidated. Recent studies indicate that many EOs and their constituents exert pharmacological properties through interactions with the GABAergic system and voltage-gated Na^+^ channels. An increasing number of studies show that: (1) many EOs used for the treatment of anxiety affect the function of the GABAergic system [13,14,15,16]; (2) many EOs with antinociceptive and anticonvulsant properties inhibit the function of neuronal voltage-gated Na^+^ channels [17]; (3) some EOs affect the function of both the GABAergic system and voltage-gated Na^+^ channels [4,18].

This review summarizes the beneficial effects of EOs and their constituents targeting the GABAergic system and neuronal voltage-gated Na^+^ channels for CNS disorders, in particular with respect to their antinociceptive, anticonvulsant, anxiolytic, and sedative effects. 

## 2. The Pharmacological Activities of EOs and the Underlying Mechanism of Their Actions

The pharmacological activities of EOs, especially antinociceptive, anticonvulsant, anti-inflammatory, anxiolytic, and sedative effects are summarized in Table 1 which shows the EOs from different plants, their pharmacological activities, and mechanism of actions.

### 2.1. EOs with Antinociceptive and Anti-Inflammatory Activities 

EOs that originate from different plants display antinociceptive and anti-inflammatory properties. Bergamot (*Citrus bergamia*) is a fruit best known for its EO used in aromatherapy to minimize symptoms of stress-induced anxiety and mild mood disorders and cancer pain [29]. The antinociceptive effect of EOs of *Salvia sclarea* (clary sage), *Thymus vulgaris* (thyme), and *Lavandula angustifolia* (true lavender), were examined in the capsaicin test [29,47]. The capsaicin test in mice is a reliable model of peripheral nociception, which produces nociceptive behavior similar to that elicited by the intraplantar injection of formalin. Among these EOs, the intraplantar injection of bergamot EO produced a significant antinociceptive effect in mice [29,47]. 

EOs from the genus *Artemisia* demonstrated antinociceptive and anti-inflammatory effects. *Artemisia dracunculus* (tarragon) has been used for the treatment of pain and gastrointestinal disturbances in Iranian traditional medicine [25]. Maham et al. [25] evaluated both central and peripheral antinociceptive activity of tarragon EO in various experimental models. It was found that the EO possesses a potent antinociceptive effect. Similarly, another EO from *Artemisia ludoviciana* was also reported to possess antinociceptive activity, which was partially mediated by the opioid system [23]. The aerial parts of *Artemisia herba-alba* are widely used to treat inflammatory disorders (colds, coughing, bronchitis, diarrhea) are infectious diseases (skin diseases, scabies, syphilis). Recent studies showed that appropriate doses of *A. herba-alba* EO displayed both antifungal and anti-inflammatory activities. Thus, the findings justified and reinforced the use of this plant in traditional medicine [22]. Abu-Darwish et al. [22] characterized the chemical composition of *A. herba-alba* EO from South Jordan and found that regular monoterpenes were predominant (39.3%) and the principal components were α- and β-thujone (27.7%). *Artemisia judaica L.* is a medicinal and aromatic plant growing in the valley bottoms of desert areas. Abu-Darwish et al. [24] studied the chemical composition and biological activities of *Artemisia judaica* EO. They found that *A. judaica* EO had antifungal and anti-inflammatory activities. For *A. judaica* EO constituents, oxygenated monoterpenes are a representative group of constituents (68.7%) in the oil with piperitone (30.4%), camphor (16.1%), and ethyl cinnamate (11.0%) as the main compounds.

*Cymbopogon citratus* is widely used in traditional medicine as an infusion or decoction, sedative, and analgesic for treating nervous disturbances [13]. An antinociceptive effect of EO from *C. citratus* has been detected in the rodent hot plate test, an experimental procedure related to central nervous system activity [48].

*Dysphania graveolens* is a traditional medicinal plant used in Mexico to treat stomach pain. The EO from the aerial parts of *D. graveolens* was recently evaluated in the hot plate and writhing tests in mice [33]. The EO produced an antinociceptive response to thermic and chemical stimuli in a mouse model [33]. 

### 2.2. EOs with Anxiolytic, Anti-Depressive, and Sedative Activities 

Popular anxiolytic oils include those of *Anthemis nobilis* (chamomile), *Salvia sclarea* (clary), *Rosmarinus officinalis* (rosemary), *Lavandula angustifolia* (lavender), and *Rosa damascena* (rose) [42]. These EOs have been used medicinally in Europe for thousands of years. In the United States, chamomile is best known as an ingredient in herbal tea preparations advertised as having mild sedation effects. The antidepressant effects of EOs of chamomile (*Anthemis nobilis*), clary (*Salvia sclarea*), rosemary (*Rosmarinus officinalis*), and lavender (*Lavandula angustifolia*) were assessed using a forced swim test (FST) in rats [42]. It was reported that among the essential oils tested, 5% (*v*/*v*) clary oil had the strongest anti-stressor effect in the FST [42]. The anti-depressive effect of clary oil in vivo could be blocked by dopaminergic antagonists, suggesting the effect is closely associated with the modulation of the dopaminergic (DA) pathway [42]. 

The fruits, leaves, and roots of *Piper guineense* have diverse medicinal uses for treating convulsion, rheumatism, and respiratory diseases in African traditional medicine [2]. The inhalation of *P. guineense* EO had significant sedative and anxiolytic effects, suggesting that the EO might induce a mild tranquilizing effect. The main compounds of *P. guineense* EO were linalool (41.8%) and 3,5-dimethoxytoluene (10.9%). These two main compounds were shown to play a major role in the sedative activity of *P. guineense* EO [2]. The EO of *Piper guineense* might exert its sedative effects partially via the GABAergic receptor system [2].

Komiya et al. [37] examined the anti-stress action of the EOs of lavender, rose, and lemon using an elevated plus-maze task, a forced swimming task, and an open field task in mice. Among the tested EOs, lemon oil had the strongest anti-stress effect in all three behavioral tasks. Unfortunately, the authors only indicate that the EOs of lavender, rose, and lemon were supplied by Soda Aromatic Co., Ltd. (Tokyo, Japan), but did not mention the botanical origin of EOs. Despite the fact that rose oil did not show a larger anti-stress effect, it has been reported that rose oil had anti-conflict effects and that the effects were not mediated by the benzodiazepine binding site of the GABA_A_ receptor complex [49]. The anxiolytic, antidepressant-like effects of lemon oil were mediated through the suppression of DA activity related to enhanced serotonergic (5-HT) neurons [37]. Lemon oil can be obtained from a species of the *Citrus* genus [37]. The EO from *Citrus aurantium* L. was reported to have an anti-stress related effect [28]. Costa et al. (2013) investigated the anxiolytic-like activity of the EO of *C. aurantium* in a light/dark box, and the antidepressant activity in a forced swim test [28]. The acute treatment with the EO showed no activity in the forced swim test, which is sensitive to antidepressants. The studies demonstrated that *C. aurantium* EO exhibited an anxiolytic-like activity which was mediated by 5-HT1A-receptors [28]. The similarity of the mechanism underlying anxiolytic-like activity suggests that lemon oil and EO from *C. aurantium* may present the same bioactive compounds or analogs which target the serotonergic system. The anxiolytic effects of *Citrus sinensis* (sweet orange) EO were evaluated on male Wistar rats in the elevated plus-maze followed by the light/dark paradigm [30]. At all doses, *C. sinensis* oil demonstrated anxiolytic activity in at least one of the tests and, at the highest dose, it presented significant effects in both animal models [30].

*Cymbopogon citratus* (DC) Stapf., commonly known as lemongrass, and *Cymbopogon winterianus* Jowitt, are widely used in traditional medicine as an infusion or decoction, sedative, and analgesic for treating nervous disturbances [13,50]. The anticonvulsant activities of the EOs from *C. winterianus* and *C. citratus* were evaluated in mice [32]. The results showed that both EOs were more active on the pentylenetetrazol-induced convulsion model, and *C. citratus* was even more efficient in increasing latency to the first convulsion and latency to death. The mechanism of action of the EOs (*C. citratus* and *C. winterianus*.) for the anticonvulsant effect was, at least in part, dependent upon GABAergic neurotransmission [32]. Costa et al. [13] investigated the anxiolytic-like activity of *C. citratus* EO in a light/dark box and marble-burying test and the antidepressant activity was investigated in forced-swimming test model in mice. The results demonstrated that acute treatment with the EO from *C. citratus* was effective against generalized anxiety disorder and epilepsy in experimental procedures in mice [51]. Studies on the underlying mechanism showed that the anxiolytic-like effect of its EO was mediated by the GABA_A_ receptor complex [13]. The main EO compounds were identified as monoterpene citral (71.29%), a mixture of the stereoisomers geranial and neral, or β-myrcene (16.5%) [13,52].

*Asarum heterotropoides* was effective in reducing anxiety and inflammation in relief of pain [53,54,55]. Park et al. (2015) reported that EO of *A. heterotropoides* effectively attenuated depression-like behavior and increased the brain expression of serotonin (5-HT) in response to six minutes of forced swimming or immobilization stress [26].

The EO of *Tugetes minutu* L. was found to have anxiogenic-like effects on T-maze and tonic immobility behavior in domestic chicks probably through the negative modulation of GABAergic function [44]. 

*Melissa officinalis* is known to have sedative, cognitive-enhancing, and relevant physiological actions. Abuhamdah et al. [38] reported that EO from *M. officinalis* reversibly inhibited GABA-induced currents in a concentration-dependent manner whereas no inhibition of NMDA- or AMPA-induced currents was noted. *Lippia alba* (Mill.) N.E. Brown is known as “false-melissa” in Brazil. This plant has been commonly used for its sedative properties, which also have been demonstrated in some rodent studies [36,56,57]. Three main chemotypes obtained from *L. alba* EO were reported and classified according to their major constituent as citral, carvone, and linalool [56]. Recently, the anesthetic effect of *L. alba* EO was demonstrated in silver catfish and the GABAergic system was involved in this effect [36]. Thus, the EO of *L. alba* is considered to be a novel natural sedative and anesthetic agent that can be potentially used in aquaculture practices due to its ability to reduce stress in fish with a consequent reduction of economic losses in fish culture [36]. Meanwhile, repeated treatment with an EO from *L. alba* displayed anxiolytic effects in the elevated T-maze mouse model [58].

Lavender (*Lavandula angustifolia*) is cultivated worldwide for its EOs, which are used in perfumes, cosmetics, food processing and, more recently, in aromatherapy products. Lavender inhalation has been used in folk medicine for the treatment of anxiety. It was reported that lavender scent reduces the anxiety state in dental patients [59]. Chioca et al. [35] reported that the EO of the plant probably exerted its anxiolytic effect through serotonergic but not GABAergic neurotransmission [35]. Lavender contains linalool and linalyl acetate as its main bioactive components [60,61]. 

Silva et al. [34] evaluated the sedative and anesthetic properties of *Hyptis mutabilis* EO and the isolated compounds from EO in silver catfish (*Rhamdia quelen*). Both the EO and the isolated compounds [(+)-1-terpinen-4-ol and (−)-globulol] showed concentration-dependent sedative and anesthetic effects.

Coriander volatile oil is prepared from *Coriander sativum*. The oil displays significant anxiolytic- and antidepressant-like effects [31]. Moreover, coriander volatile oil decreased the catalase activity and increased glutathione level in the hippocampus [31]. The multiple exposures to coriander volatile oil can be useful as a means to counteract anxiety, depression, and oxidative stress in Alzheimer’s disease conditions. The GC-MS and GC-FID analyses determined the main volatile component of the coriander volatile oil sample to be linalool (69.4%), which is probably the responsible constituent for the observed anxiolytic- and antidepressant-like effects. This assumption was confirmed by the fact that inhaling linalool rich EOs can be useful as a means to counteract anxiety [35,62]. Other minor constituents of coriander volatile oil are α-pinene (3.0%–7.0%) and γ-terpinene (1.5–8.0%) [31].

It was reported that the EO of rhizome and leaf of *Acorus gramineus* displayed sedative and anticonvulsant properties and also anti-oxidative activity after fragrance inhalation [20]. Koo et al. [20] reported that pre-inhalation of the EO of *Acorus gramineus* markedly delayed the appearance of pentylenetetrazole-induced convulsion. Moreover, fragrance inhalation progressively prolonged the pentobarbital-induced sleeping time as the inhalation time was lengthened. This sedative effect after inhalation or oral administration of *A. gramineus* EO suggests that this oil may act on the CNS via the GABAergic system. The main components in the EO are β-asarone (40.3%), euasarone (17.0%) and α-euasarone (12.3%) [20]. 

Valerian (*Valerian officinalis* L), a common name given to the crude drug consisting of the underground organs of species of the genus *Valeriana* (Valerianaceae), demonstrated sedative activity [46]. The constituents of the volatile oil of valerian are very variable due to population differences in genetics and environmental factors. The major constituents include the monoterpene bornyl acetate and the sesquiterpene valerenic acid, which is characteristic of the species, in addition to other types of sesquiterpene. The non-volatile monoterpenes known as valepotriates were first isolated in 1966 and contribute to the overall activity by possessing sedative activity based on the effects on the CNS [46]. Valepotriates consist of the furanopyranoid monoterpene skeleton commonly found in the glycosylated forms [46].

EO from *Cananga odorata* (ylang-ylang EO, YYO) is usually used in reducing blood pressure, improving cognitive functioning in aromatherapy in humans. It was recently reported that YYO displayed anxiolytic effects on anxiety behaviors [63]. YYO and the three main constituents of YYO (benzyl benzoate, linalool, and benzyl alcohol) increased the time that mice visited the open arms and a lightbox area in the elevated plus-maze and light-dark box tests after acute and chronic YYO exposures [63]. YYO and its major constituent benzyl benzoate might act on the serotonergic and dopaminergic pathways [63].

A mixture of different EOs was reported to have inhibitory effects on CNS. The central nervous system inhibitory effects of the EO from *SuHeXiang Wan* (Storax pill) on fragrance inhalation (aromatherapy) were evaluated. *SuHeXiang Wan* consists of 15 crude herbs, among them *Liquidambar orientalis*, *Saussurea lappa*, *Aquilaria agallocha*, *Santalum album*, *Boswellia carterii*, *Eugenia caryophyllata*, *Styrax benzoin*, *Dryobalanops aromatica*, and *Cyperus rotundus*. All of them have the term “Xiang” (fragrance) in their Chinese plant names. The fragrance inhalation of the EO from *SuHeXiang Wan* progressively prolonged the pentobarbital-induced sleeping time and inhibited brain lipid peroxidation to which the anticonvulsive action is attributed [15]. The studies confirm that the inhibitory effects of the EO of *SuHeXiang Wan* on the CNS were mediated by the GABAergic system [15]. Similarly, the perfume, which is a mixture of different EOs, affects the frame of the human mind. The effects of the perfume and phytoncid on GABA_A_ receptors expressed in *Xenopus* oocytes were studied [40]. The chemical constituents in the perfume such as hinokitiol, pinene, eugenol, citronellol, and citronellal potentiated the GABA_A_ receptor expressed in *Xenopus* oocytes. These results suggest that the GABAergic system is a common target of EOs.

In addition to acting on the GABAergic system, the pharmacological effects of EOs may be mediated through other receptor systems. Different species of *Achillea* are used in folk medicine as sedatives, anti-inflammatory agents, analgesic agents, and anthelmintic (antiparasitic) agents. Majnooni et al. [19] reported that the EO of *A. wilhelmsii* had anxiolytic effects which were probably not mediated through GABA and opioid receptors. Gas chromatography/mass spectrometry (GC/MS) analysis of the EO showed that the main compounds of the oil were *p*-ocimen (23%), 1, 8-cineole (20.8%), and carvone (19.13%). *Pistacia integerrima* Stewart ex Brandis galls are used in Indian ethnomedicine for their anti-asthmatic, sedative, and spasmolytic properties. The EO of the plant had relaxant and spasmolytic effects which may be mediated by modulating β-adrenoceptors and calcium channels [41].

### 2.3. EOs with Anticonvulsant and Other Pharmacological Properties

The major chemical components detected in the *Aloysia citrodora* EOs, derived from dried and fresh leaves, included limonene, geranial, neral, 1,8-cineole, curcumene, spathulenol, and caryophyllene oxide, respectively. *A. citrodora* leaf EO inhibited [^3^H] nicotine binding to well-washed rat forebrain membranes, and increased iron-chelation in vitro [21]. *A. citrodora* EO displays the effective antioxidant, radical-scavenging activities, and significant protective properties [21].

Raza et al. [39] investigated the antiepileptic effects of *Nigella sativa* seed volatile oil and the main components of the oil (that is, thymoquinone, α-pinene and *p*-cymene) using pentylenetetrazole (PTZ) and maximal electroshock (MES)-induced convulsions [39]. The volatile oil protected mice effectively against PTZ-induced convulsions that may be attributed mainly to its content of thymoquinone and *p*-cymene and to a lesser extent, α-pinene. Volatile oil and its component *p*-cymene effectively suppressed convulsions induced by MES. Their studies suggest that picrotoxin and bicuculline-sensitive GABA receptors mediate an increase in the GABAergic response. 

## 3. The Pharmacological Properties of Constituents Isolated from EOs and the Underlying Mechanisms of Action

Natural products have played pivotal roles in neuropharmacology due to their potent and selective targeting of specific biochemical pathways and receptors, and are highly useful as probe substances and therapeutic leads. An increasing amount of evidence shows that EOs present potent bioactive constituents targeting different therapeutic targets [4,5]. 

Terpenoids and phenylpropanoid derivatives are the main constituents of EOs [3]. The most common terpenoids found in EOs are the monoterpenes and the sesquiterpenes. There is increasing interest in the pharmacological actions of the constituents found in EOs, especially terpenes [3]. Here, we summarize the pharmacological properties of the constituents isolated from EOs and the mechanisms underlying their actions. Table 2 is a summary of pharmacological properties of EO constituents from different plants and the underlying mechanisms of their effects.

### 3.1. Analgesic and Anticonvulsant Properties

The main components of the EOs of aromatic plants, terpenes, and terpenoids are considered important agents in the food industry and for medicinal use. The constituents of the EOs of aromatic plants are emerging therapeutic resources for developing new drugs to treat chronic pain [95]. An increasing number of terpenes and terpenoids are found to have analgesic anti-inflammatory properties.

#### 3.1.1. Terpenoids with Analgesic Properties Targeting Na^+^ and TRP Channels

1,8-Cineole is a terpenoid present in many EOs of plants with several pharmacological and biological effects, including antinociceptive, smooth muscle relaxant, and ion channel action. Additionally, 1,8-cineole blocked action potentials, thereby reducing the excitability of peripheral neurons [96,97]. Ferreira-da-Silva et al. [64] demonstrated that 1,8-cineole directly affects Na^+^ channels of the superior cervical ganglion neurons by modifying several gating parameters that are likely to be the major cause of excitability blockade. Eucalyptol reduced the excitability of rat sciatic nerve and superior cervical ganglion neurons [96,97]. On the other hand, Zeraatpisheh and Vatanparast [65] reported that 1,8-cineole (eucalyptol) induced hyperexcitability and epileptiform activity in snail neurons, which is most likely mediated through the direct inhibitory action on the potassium channels.

**Figure 1:**
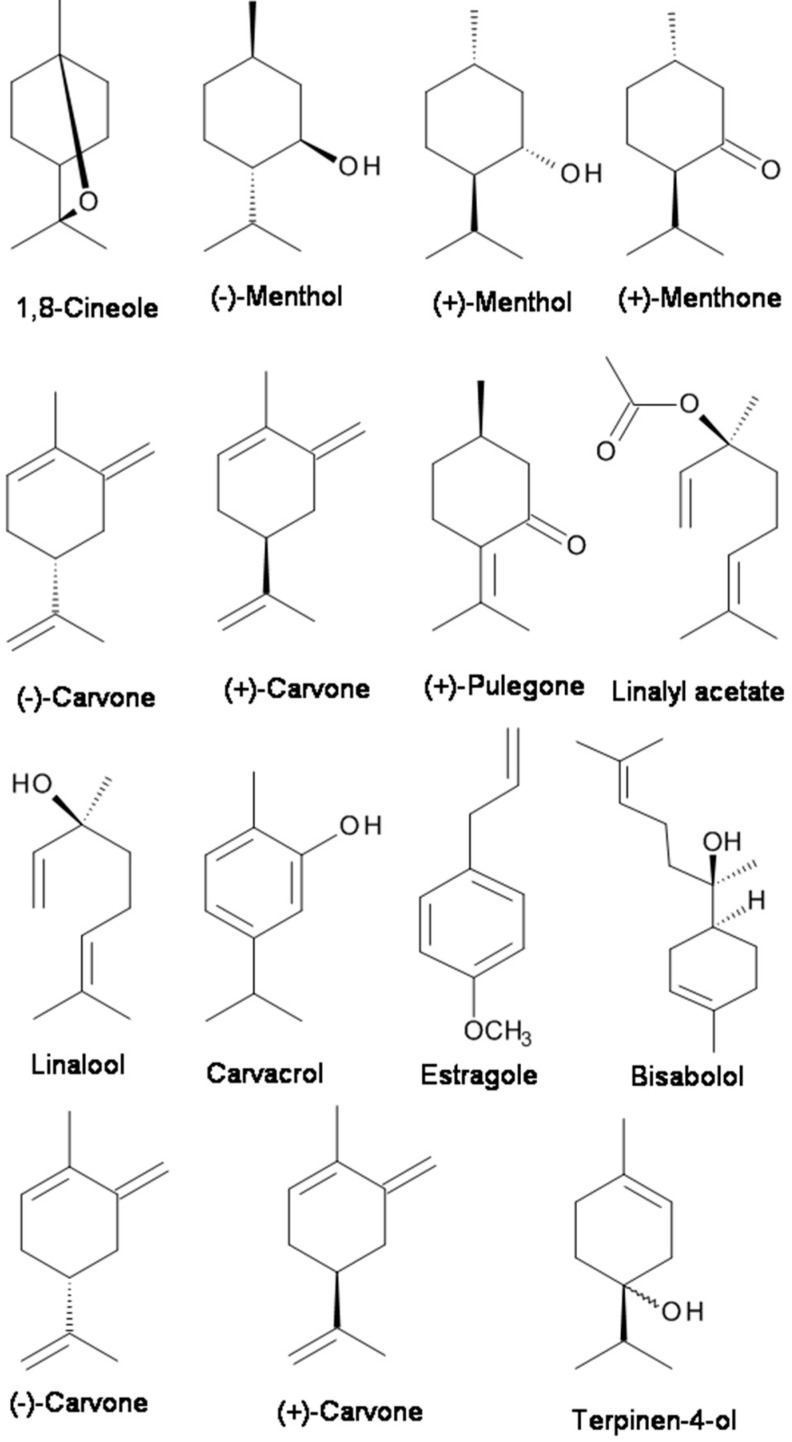
The chemical structures of terpenes with analgesic properties targeting the Na^+^ and transient receptor potential (TRP) channels.

Menthol (2-isopropyl-5-methylcyclohexanol), which is contained in peppermint or other mint oils, is well-known to induce analgesia by activating the transient receptor potential (TRP) melastatin-8 (TRPM8) channels [86]. By recording the compound action potentials (CAPs), Kawasaki et al. [86] examined the effects of menthol and its related compounds on CAP peak amplitude. (−)-Menthol and (+)-menthol concentration-dependently reduced the CAP peak amplitude. (−)-Menthone, (+)-menthone, (−)-carvone, (+)-carvone, (+)-carveol, and (+)-pulegone inhibited CAPs with extents similar to that of menthol (see Figure 1) [86]. They found that menthol and its related compounds reduce CAP peak amplitude in a manner specifically related to their chemical structures and that menthol activity is not mediated by TRPM8 channels [86].

Terpenoids (for example, 1,8-cineole, linalool and linalyl acetate) are the main chemical components of EOs of the *Lavandula* genus, which includes lavender (*Lavandula angustifolia*), lavandin (*L. angustifolia hybrid L. latifolia*), and bergamot (*Citrus bergamia*). Lavandin is actually a hybrid created from true lavender (*L. augustifolia*) and spike lavender (*L. latifolia)*. Even though the EO containing linalool and/or linalyl acetate demonstrated an antinociceptive effect [47], the main constituents of the EO, linalool and linalyl acetate (Figure 1), were much more potent than the bergamot EO or other EOs in inhibiting the response to intraplantar capsaicin [29,47,98]. Linalool has been the compound mostly linked to the anxiolytic effect of lavender [35,60,61,62,99,100,101]. Moreover, Takahashi et al. [60] showed that the anxiolytic-like effect of linalool in mice tested in the elevated plus-maze was potentiated by linalyl acetate. Also, it was reported that linalool displayed depressant effects on the central nervous system and olfactory receptors [85]. Leal-Cardoso et al. [85] investigated the effects of linalool on the excitability of peripheral neurons of the somatic sensory system. They found that linalool acts on the somatic sensory system with local anesthetic properties since it blocked the action potential by acting on voltage-dependent Na^+^ channels. 

Carvacrol (5-isopropyl-2-methylphenol) (see Figure 1) is a monoterpenic phenol present in the EOs of many plants, especially in the genera *Origanum* and *Thymus* [81]. It is the major constituent of the EO fraction of oregano and thyme. Carvacrol has attracted attention because of its beneficial biological activities, especially analgesic activity. Gonçalves et al. [45] characterized the constituents of *Thymus capitatus* EO and evaluated their antinociceptive activity by in vivo and in vitro procedures. The EO of *T. capitatus* presented 33 components, mainly monoterpenes and sesquiterpenes, and carvacrol (80%) was its major constituent. EO of *T. capitatus* dose-dependently decreased the CAP amplitude. Such activity was presumably mediated through voltage-gated Na^+^ channels [45]. Melo et al. [81] examined the behavioral effects of carvacrol using elevated plus-maze (EPM), open field, rotarod, and barbiturate-induced sleeping time tests in mice. The results indicate that carvacrol had anxiolytic effects in the plus-maze test which are not influenced by the locomotor activity in the open-field test [81]. The mechanism underlying the anesthetic and analgesic effects is based on a reduction in neuronal excitability and voltage-gated Na^+^ channel inhibition in peripheral neurons [79]. The hypothesis of the underlying mechanism was supported by the finding that carvacrol could reduce the total voltage-gated Na^+^ current and tetrodotoxin-resistant (TTX-R) Na^+^ current component in a concentration-dependent manner in isolated dorsal root ganglion neurons [80]. In addition to analgesic activity mediated through the blockade of Na^+^ channels [45,80], Trailović et al. [78] tested the effects of different concentrations of carvacrol on isolated tissues of the large pig nematode *Ascaris suum*. The somatic muscle flaps were used for contraction assays and for electrophysiological investigations. The inhibitory effect of carvacrol on contractions, the inhibition of depolarizations caused by acetylcholine (ACh), and the reduction of conductance changes directly point to an interaction with nicotinic ACh receptors in *A. suum*.

Bisabolol, or more formally α-(−)-bisabolol or also known as levomenol, is a natural monocyclic sesquiterpene alcohol (Figure 1). It is a primary constituent of the essential oil from *Matricaria chamomilla* [74]. Bisabolol is known to have anti-irritant, anti-inflammatory, and anti-microbial properties. Recently, it was reported that bisabolol demonstrated an antinociceptive-like effect which could be associated with decreased peripheral nerve excitability [74]. The decreased nervous excitability elicited by α-(−)-bisabolol might be caused by an irreversible blockade of voltage-dependent Na^+^ channels [74].

Estragole, a volatile terpenoid, is the primary constituent of EO of tarragon (comprising 60–75%). It has several pharmacological and biological activities, including antioxidant, anxiolytic, and antimicrobial activities [82]. The mechanism of action of estragole on neuronal excitability was recently investigated. The intact and dissociated dorsal root ganglion neurons of rats were used to record action potential and Na^+^ currents with intracellular and patch-clamp techniques, respectively. It was found that estragole blocked neuronal excitability by direct inhibition of Na^+^ channel conductance activation. 

Carvone (*p*-mentha-6,8-dien-2-one) (Figure 1) is a chiral monoterpene ketone that is present in *Mentha spicata* (Spearmint) and *Carum carvi* (Caraway) EOs and has been shown to have anticonvulsant [102], antinociceptive [103] and anxiolytic-like effects in the elevated T maze [58]. Both (*R*)-(−)-carvone and (*S*)-(+)-carvone decreased peripheral nerve activity, likely through the blockade of voltage-gated Na^+^ channels [77,104]. (*S*)-(+)-Carvone appears to have anticonvulsant activity against pentylenetetrazole- and picrotoxin-induced seizures [102]. In vivo, both (*R*)-(−)-carvone and (*S*)-(+)-carvone display antimanic-like effects in two mouse models [77].

Kang et al. [105] examined the effects of (−)-carvone and its stereoisomer (+)-carvone (in caraway) on glutamatergic spontaneous excitatory transmission in SG neurons of adult rat spinal cord slices by using the whole-cell patch-clamp technique. They found that (−)-carvone and (+)-carvone activate TRPV1 and TRPA1 channels, respectively, resulting in an increase in spontaneous L-glutamate release onto SG neurons, with almost the same efficacy [105].

Terpinen-4-ol (Figure 1) is a monoterpenoid alcoholic component of EOs obtained from several aromatic plants. Nóbrega et al. [90] investigated the psychopharmacological and electrophysiological activities of Terpinen-4-ol in male Swiss mice and Wistar rats [90]. Terpinen-4-ol (*i.p.*) inhibited pentylenetetrazol-(PTZ-) induced seizures, indicating anticonvulsant effects. The anticonvulsant action exerted by Terpinen-4-ol involved the GABAergic system but did not bind to the benzodiazepine-binding site of GABA_A_ receptors [90]. Furthermore, the electrophysiological results show that terpinen-4-ol decreased a sodium current through voltage-dependent sodium channels [103]. Thus, its anticonvulsant effect may be related to changes in neuronal excitability based on the modulation of both the GABAergic system and Na^+^ channels. 

#### 3.1.2. Terpenes with Analgesic and Anticonvulsant Properties Targeting GABA_A_ Receptors

Hossain et al. [27] electrophysiologically tested the effect of fragrant compounds in oolong tea on GABA_A_ receptors which were expressed in *Xenopus oocytes*. Oolong tea is a traditional Chinese tea that is made by withering the plants with strong sun exposure and oxidation. *cis*-Jasmone, jasmine lactone, linalool oxide, and methyl jasmonate significantly potentiated the response to GABA. The inhalation of 0.1% cis-jasmone or methyl jasmonate significantly increased the sleeping time of mice induced by pentobarbital. The results suggest that these fragrant compounds could be absorbed by the brain and thereby potentiated the GABA_A_ receptor response to exerting a tranquilizing effect on the brain [27].

Nerolidol is an acyclic sesquiterpene found as a major constituent of several EOs such as *Piper claussenianum* and *Burchardia umbellata* [89]. Fonsêca et al. [89] evaluated the antinociceptive activity of nerolidol using the acetic acid-induced writhing test, the formalin test, and the hot-plate test. The results showed that nerolidol had antinociceptive activities in chemical nociception models (acetic acid-induced writhing test and formalin test), but not in the thermal nociception model (hotplate test). The analgesic activity of nerolidol is possibly related to the GABAergic system, and not to the opioidergic system or to ATP-sensitive K^+^-channels [89]. 

**Figure 2:**
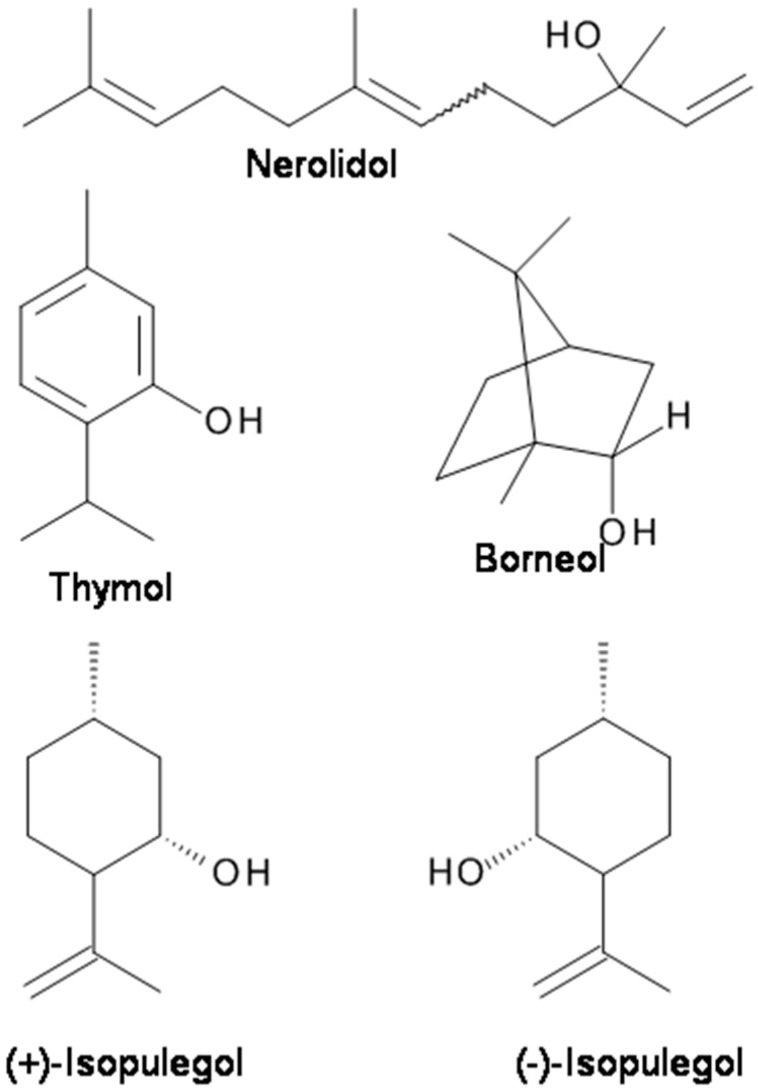
The chemical structures of terpenes with analgesic and anticonvulsant properties targeting GABA_A_ receptors.

Natural borneol is (+)-borneol (Figure 2). (+)-Borneol is a bicyclic monoterpene present in the EOs of numerous medicinal plants, including valerian (*Valeriana officinalis*), chamomile (*Matricaria chamomilla*), and lavender (*Lavandula officinalis*). It is used for analgesia and anesthesia in traditional Chinese medicine [69]. (+)-Borneol has remarkable anti-hyperalgesic effects on neuropathic and inflammatory pain in animal models [69]. The results suggest that (+)-borneol may ameliorate mechanical hyperalgesia by enhancing GABA_A_R-mediated GABAergic transmission in the spinal cord and could serve as a therapeutic for chronic pain [69]. Granger et al. [14] reported that both (+)-borneol and its enantiomer (−)-borneol directly potentiate GABA activity at recombinant human α_1_β_2_γ_2L_ GABA_A_ receptors are expressed in *Xenopus laevis* oocytes. Both (+)-borneol and (−)-borneol demonstrated a highly efficacious positive modulating action at GABA_A_ receptors [14]. In vivo, (+)-borneol displays significant antinociceptive effect in models of chronic pain in mice without producing a motor deficit. These findings suggest that borneol may ameliorate mechanical hyperalgesia by enhancing GABA_A_R-mediated GABAergic transmission in the spinal cord and could serve as a therapeutic for chronic pain.

Thymol is a monoterpenoid monocyclic phenolic compound. It is the main component of the EO of *Thymus vulgaris* (Lamiaceae). The main therapeutic application of thymol is in dental preparations to kill odor-producing bacteria and has various actions including antinociception and nerve conduction inhibition [92,93,106]. Thymol has been reported to activate transient receptor potential (TRP) channels expressed in heterologous cells [107] and act as a positive allosteric modulator of human GABA_A_ receptors and a homo-oligomeric GABA receptor from Drosophila melanogaster [93]. Thymol displayed inhibition on spontaneous excitatory transmission in adult rat spinal substantia gelatinosa (SG) neurons, suggesting that thymol increases the spontaneous release of l-glutamate onto the neurons by activating TRPA1 channels while producing an outward current without TRP activation [92]. Considering that the substantia gelatinosa plays a pivotal role in modulating nociceptive transmission from the periphery, the actions of thymol could contribute to at least a part of its antinociceptive effect [92].

Isopulegol (*p*-menth-8-en-3-ol) (Figure 2), a monoterpene alcohol of the menthane family, is present in the EOs of various plants species, such as *Eucalyptus citriodora* and *Zanthoxylum schinifolium* [84]. It was found that similar to diazepam, isopulegol significantly prolonged the latency for convulsions and mortality of mice in a PTZ-induced convulsion animal model [84]. The results suggest that the anticonvulsant effects of isopulegol against PTZ-induced convulsions are possibly related to positive modulation of benzodiazepine-sensitive GABA_A_ receptors [84].

#### 3.1.3. Phenylpropanoid Derivative Constituents with Analgesic Properties and the Mechanisms of Action

**Figure 3:**
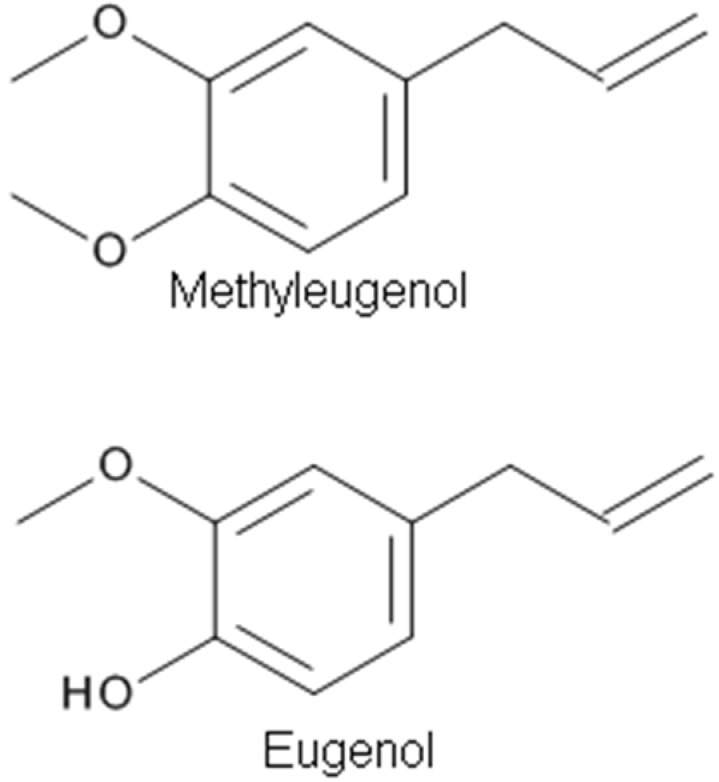
The chemical structures of phenylpropanoid derivatives with analgesic properties.

Methyleugenol (4-allyl-1,2-dimethoxybenzene) (Figure 3), a phenylpropanoid derivative, is a natural constituent isolated from EOs of many plants, such as Chinese herb *Asari Radix et Rhizoma*, having multiple biological effects including anticonvulsant, antinociceptive, and anesthetic activities. The anesthetic property of methyleugenol has been demonstrated by a loss of the righting reflex and decreased sensitivity to a tail pinch in rats and mice, and a loss of the corneal reflex in rabbits [5]. The antinociceptive and anesthetic effects of methyleugenol resulted from the inhibitory action of methyleugenol on peripheral Na^+^ channels [5]. Yano et al. [88] tested the effects of methyleugenol on antinociception using the formalin test in mice in vivo. They found that the antinociceptive effect of methyleugenol on the second phase of formalin-induced pain might be due to the inhibition of NMDA receptor-mediated hyperalgesia via GABA_A_ receptors. Ding et al. [87] tested the action of methyleugenol on GABA_A_ receptors. At lower concentrations (~30 μM), methyleugenol significantly potentiated GABA-induced currents in cultured hippocampal neurons [87]. Similarly, methyleugenol potentiated GABA-induced currents mediated by recombinant α_1_β_2_γ_2_ or α_5_β_2_γ_2_ GABA_A_Rs in human embryonic kidney (HEK) cells. In addition to the blockade of Na^+^ channels [5], this study adds GABA_A_R activation to the list of molecular targets of methyleugenol [87].

Eugenol (4-allyl-2-methoxyphenol) (Figure 3), an aromatic phenylpropanoid molecule found in plants including *Syzygium aromaticum* (Clove), has been used in medicine to relieve pain [43]. The EO of clove, which is made from the aromatic flower buds of a tree in the family Myrtaceae, *Syzygium aromaticum*, is known as an important weak local anesthetic for dental pain [17,43]. Eugenol possesses analgesic effects that may be related to the inhibition of voltage-dependent Na^+^ channels and/or to the activation of TRPV1 receptors or both. Moreira-Lobo et al. [17] reported that eugenol inhibited action potentials and modified the excitability of the rat sciatic nerve and superior cervical ganglion neurons. Huang et al. [43] reported that eugenol reduced the firing of neuronal action potentials and hyperexcitability through a synergistic blocking effect of Na^+^ currents. Recently, Vatanparast et al. [83] studied the effects of eugenol on the excitability of central neurons of the land snail *Caucasotachea atrolabiata* and the underlying ionic mechanisms. It was found that a low concentration of eugenol could have antiepileptic properties, while at a higher concentration, it induced epileptiform activity. The dose-dependent inhibition of the ionic currents underlying the rising and falling phases of the action potential seems to be relevant to the eugenol suppressant and excitatory actions, respectively [83].

### 3.2. Anxiolytic, Sedative, and Anti-Depressive Properties 

EOs with anxiolytic, anti-depressive, and sedative properties usually interact with the GABAergic system. Some constituents contained in the EOs might act on the GABAergic system to exert their pharmacological effects.

#### 3.2.1. Terpenes with Anxiolytic and Sedative Properties Targeting the GABAergic System

An increasing number of terpenes have been reported to have anxiolytic and sedative activities. A promising line of research on terpenes has attributed the sedative and anxiolytic effects to the modulation of GABA_A_ receptor function [16].

**Figure 4:**
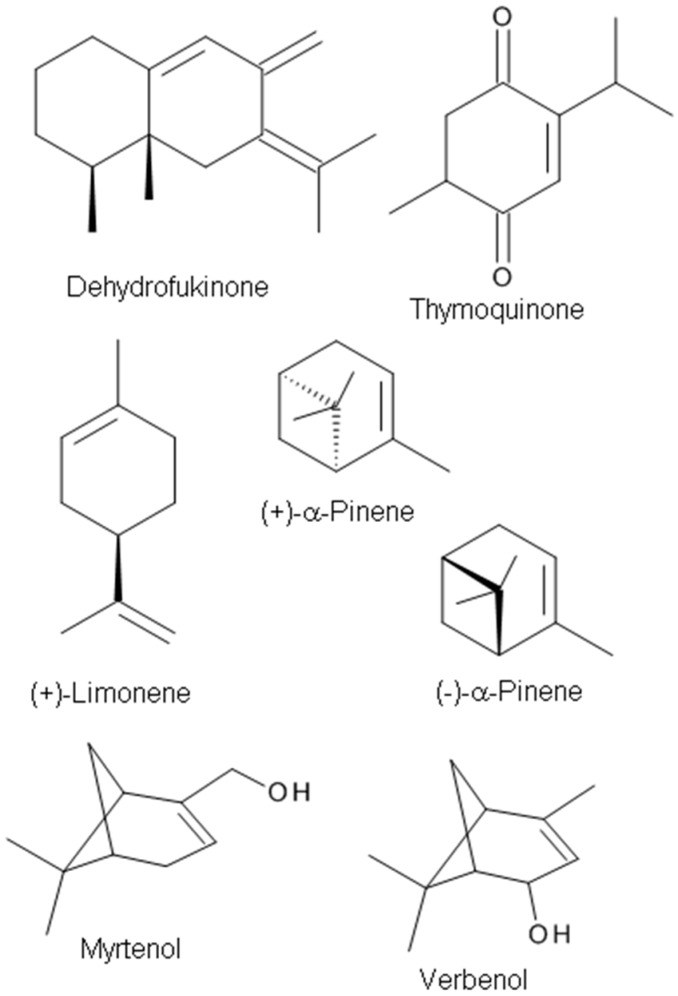
The chemical structures of terpenes with anxiolytic targeting GABA_A_ receptors.

The monoterpene (+)-limonene (Figure 4), a chemical constituent of various bioactive EOs, is the major chemical component (58.4%) of the *Citrus aurantifolia* EO [108]. The anxiolytic-like properties of *Citrus* EOs from *C. aurantium* L. and sweet orange aroma have been demonstrated in rodent models [30,109]. Bergamot (*C. bergamia*) is a fruit best known for its EO. The oil is used in aromatherapy to minimize symptoms of stress-induced anxiety and mild mood disorders and cancer pain [29]. The anxiolytic-like effects of (+)-limonene were reported in an elevated maze model of anxiety in mice. However, the molecular target protein of the compound was not studied in that report [72]. s-Limonene is a component of lemon EO. The studies on the anti-stress effect of s-Limonene suggest that the effect may be mediated through the GABAergic system [71]. 

(+)-Dehydrofukinone (Figure 4), also known as dihydrokaranone, is an eremophilane-type sesquiterpenoid ketone isolated from *Nectandra grandiflora* Ness (Lauraceae) EO. Recent behavioral studies have indicated that dehydrofukinone has sedative and anesthetic properties mediated by GABAergic mechanisms in fish [70], and induces sedation and anesthesia by modulation of GABA_A_ receptors in a mouse model [73], suggesting that the natural compound (+)-dehydrofukinone has therapeutic potential as a suppressor of neuronal excitability.

Thymoquinone (Figure 4), a major constituent of *Nigella sativa* seeds EO (27.6–57.0%), exhibits anticonvulsant activity in the PTZ-induced seizure model [94]. The anticonvulsant effects are probably mediated through an opioid receptor-mediated increase in GABAergic tone. Gilhotra and Dhingra [110] investigated the role of GABAergic and nitriergic modulation in the antianxiety effect of thymoquinone. Thymoquinone (20 mg/kg) showed anxiolytic effects with a significant decrease in plasma nitrite and the reversal of decreased brain GABA content in stressed mice [110].

Pine EO was reported to have anti-inflammatory, antimicrobial, analgesic, and anti-stress effects [75,111,112]. The main components in the oil are α- and β-pinene, 3-carene, limonene, and terpinene [113]. α-Pinene [2,6,6,-trimethylbicyclo(3.1.1)-2-hept-2-ene] (Figure 3), a major monoterpene of pine EOs, shows anxiolytic and hypnotic effects upon inhaled administration and a sleep enhancing property through a direct binding to GABA_A_ receptors by acting as a partial modulator of GABA_A_ receptors [75]. 

Sideritis plants and their extracts have been used in traditional medicine as sedatives, anxiolytics, and anticonvulsant agents. Kessler et al. [114] demonstrated that volatile aroma substances in sideritis tea extracts have a powerful modulatory effect on synaptic α_1_/β_2_GABA_A_Rs (with or without γ_2_ subunits) in a heterologous expression system. Pinenes are the most prevalent of the volatile aroma components in Siderites extracts and the pinene metabolites myrtenol and verbenol (Figure 4) have been identified as the most potent positive allosteric modulators of synaptic-type GABA_A_ receptors composed of α_1_β_2_ and α_1_β_2_γ_2_ subunits [114]. van Brederode et al. [16] examined the two terpenoids, myrtenol, and verbenol and found augmented tonic GABA currents mediated by extrasynaptic GABA_A_ receptors containing the δ subunit. It was reported that terpenoid substances potentiated the response to GABA in HEK293 cells transfected with GABA_A_Rs composed of subunits that typically mediate tonic GABA inhibition in the brain. Their results suggest that myrtenol and verbenol act as positive allosteric modulators at synaptic and extrasynaptic GABA_A_ receptors, thereby augmenting phasic and tonic GABAergic inhibition [16]. 

#### 3.2.2. Terpenes with Other Pharmacological Properties

β-Citronellol is an alcoholic monoterpene found in EOs such as *Cymbopogon citrates*, a plant with antihypertensive properties. Vasconcelos et al. [76] assessed its pharmacological effects on the contractility of rat trachea. β-Citronellol exerted inhibitory effects on rat tracheal rings, with predominant effects on contractions that increased Ca^2+^ inflow towards the cytosol by voltage-gated pathways [76]. β-Citronellol antagonized transmembrane Ca^2+^ influx from the extracellular milieu to produce myorelaxant actions [76].

#### 3.2.3. Non-Terpene Constituents with Anticonvulsant, Anxiolytic Properties, and Their Underlying Mechanisms

**Figure 5:**
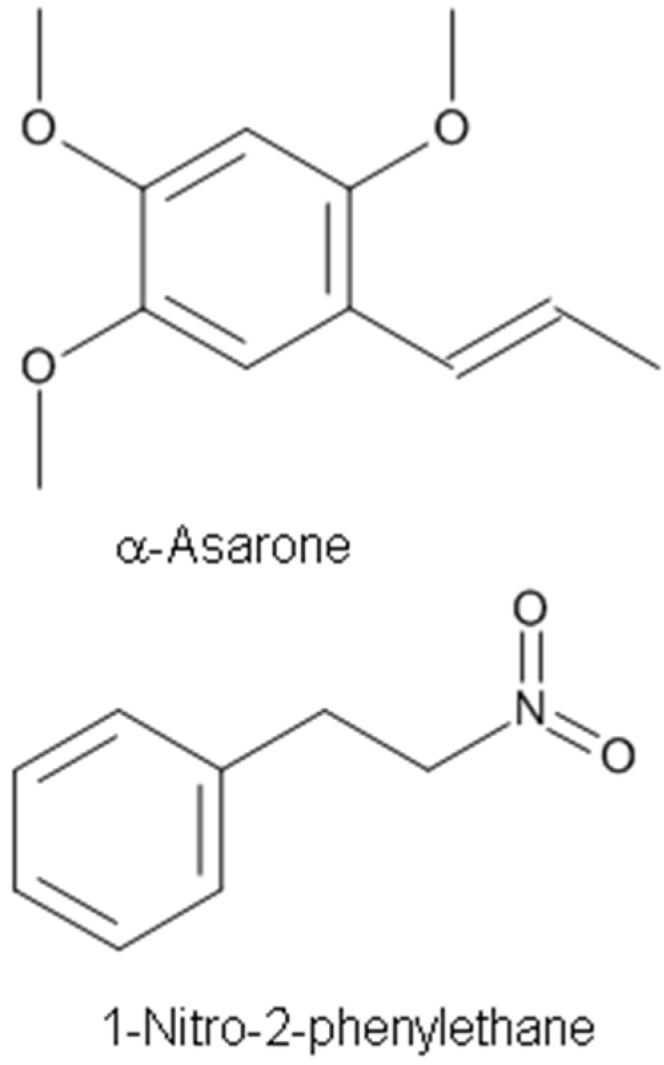
The non-terpene constituents with anticonvulsant and anxiolytic activities.

Alpha(α)-asarone (Figure 5), a major effective component isolated from the Chinese medicinal herb *Acorus tatarinowii*, is clinically used as a medication for treating epilepsy, cough, bronchitis, and asthma. Huang et al. [54] evaluated the action of α-asarone on the excitability of rat hippocampal neurons in culture and on the epileptic activity induced by pentylenetetrazole or kainite injection in vivo. They found that α-asarone inhibits the activity of hippocampal neurons and produces an antiepileptic effect in the central nervous system through enhancing tonic GABAergic inhibition [54]. Using whole-cell patch-clamp recording, α-asarone was reported to inhibit the spontaneous firing of output neurons, mitral cells, in a mouse olfactory bulb brain slice preparation [4]. 

It was reported that α-asarone alleviates epilepsy by modulating GABA_A_ receptors and inhibiting neuronal Na^+^ channels [4,54]. While many other compounds like borneol were reported to act as positive allosteric GABA_A_ receptor agonists to exert anxiolytic-like effects [14,16,102], recent studies demonstrated that α-asarone acted as both a positive allesteric GABA_A_ receptor agonist as well as a neuronal Na^+^ channel blocker [4,5]. 

The compound 1-Nitro-2-phenylethane (Figure 5) is the first nitro compound isolated from plants and is thought to be responsible for the plant’s cinnamon scent [67]. It is the main constituent of the EO of *Aniba canelilla* [67]. The mechanisms underlying the vasorelaxant effects of the EO of *Aniba canelilla* (EOAC) and its main constituent 1-nitro-2-phenylethane (NP) were investigated in the isolated superior mesenteric artery from spontaneously hypertensive rats (SHRs) [68]. Both EOAC and NP relaxed the contraction evoked by phorbol dibutyrate. Thus, it appears that NP is the active principal component of EOAC [67,68]. The vasorelaxation appears to be mediated through the inhibition of contractile events that are independent of Ca^2+^ influx from the extracellular milieu [67]. 

The EO of *Dennettia tripetala* G. Baker (Annonaceae) demonstrated significant analgesic, anti-inflammatory, hypothermic, sedative, muscle relaxant, and central nervous system depressant activities [115]. The EO of *D. tripetala* contains several compounds including 1-nitro-2-phenylethane (80%), β-eudesmol andnerolidol (4%), 1-linalool (11%), β-caryophyllene, and β-humuline. The compound 1-Nitro-2-phenylethane obtained from the oil of *D. tripetala* exhibited dose-dependent hypnotic, anticonvulsant and anxiolytic effects, and is the compound largely responsible for the neuropharmacological effects of the oil [66].

### 3.3. Terpenes with Convulsive Activities Acting as GABA_A_ Receptor Antagonists 

**Figure 6:**
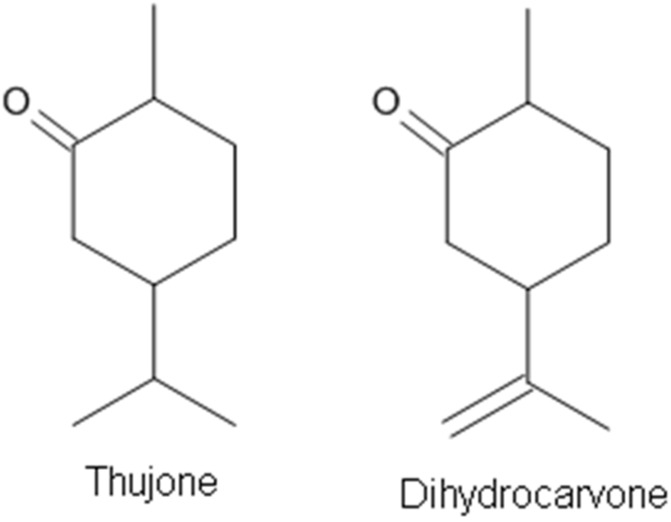
The terpenes acting on GABA_A_ receptors as antagonists.

Most components of EOs act as GABA receptor agonists. Only a few compounds from EOs have been demonstrated to be GABA_A_ receptor antagonists. Thujone (Figure 6), a cyclic monoterpenic ketone, is an active ingredient of wormwood oil and some other herbal medicines [91,116]. It is known that thujone is specifically a GABA_A_ receptor antagonist and, by inhibiting GABA receptor activation, may make neurons fire more easily, causing muscle spasms and convulsions [116,117]. Dihydrocarvone (Figure 6) is present in oils of the caraway plant and is used for its fragrance as flavoring and for medicinal purposes. Dihydrocarvone was recently found to act as a negative allosteric modulator of this receptor [91].

## 4. Conclusions

Based on the findings discussed above, it is clear that natural EOs demonstrate many neuro-pharmacological properties, such as anti-nociceptive, anti-inflammatory, anxiolytic, anti-depressive, and sedative properties. An increasing number of studies show that two inhibitory systems, the GABAergic system and the neuronal voltage-gated Na^+^ channels, are most likely involved in such pharmacological effects. EOs are emerging as a promising source for modulation of the GABAergic system and Na^+^ channels. The neuro-pharmacological activities and the underlying mechanisms of many constituents obtained from EOs have been reviewed. Most of the above-mentioned constituents are reported to have either anti-nociceptive or anxiolytic effects through activating the GABAergic system and by inhibiting neuronal Na^+^ channels. Only a few constituents have been reported to antagonize GABA receptors. The perspective compounds targeting the GABAergic system and/or voltage-gated Na^+^ channels could serve as better candidates or pharmacophores during new drug development to control pain and anxiety syndromes. 

## Figures and Tables

**Table 1:** The summary of essential oils from different plants, their pharmacological properties, and mechanism of actions.

EO Botanical Origins	Administration	Pharmacological Effects	Mechanism of Actions	Authors/Year/Ref.
*Achillea Wilhelmsii* C. Koch	*i.p.*	anxiolytic effects	not probably mediated through GABA and opioid receptors	Majnooni et al., 2013 [19]
*Acorus gramineus* Rhizoma	*INH*; *p.o.*	pentylenetetrazole-induced convulsion; sedative effect; CNS inhibitory effects	increased GABA level; decreased GABA transaminase	Koo et al., 2003 [20]
*Acorus tatarinowii* Schott		analgesic effects	inhibited Na^+^ channels	Moreira-Lobo et al., 2010 [17]
*Aloysia citrodora* Palau	in vitro	effective antioxidant, radical-scavenging activities, and neuronal protection	inhibited [^3^H] nicotine binding	Abuhamdah et al., 2015 [21]
*Artemisia herba-alba*	in vitro	antifungal and anti-inflammatory activities	N/A	Abu-Darwish et al., 2015 [22]
*Artemisia ludoviciana*	*i.p.*	antinociceptive activity	partially mediated by the opioid system	Anaya-Eugenio et al., 2016 [23]
*Artemisia judaica*	in vitro	antifungal and anti-inflammatory activities	N/A	Abu-Darwish et al., 2016 [24]
*Artemisia dracunculus*	*i.p.*	peripheral and central antinociceptive activity	N/A	Maham et al., 2014 [25]
*Asarum heterotropoides*	*INH*	decreased depression-like behaviors	N/A	Park et al., 2015 [26]
*Camellia sinensis*	*INH*	increased sleeping time	potentiated GABA_A_ receptors	Hossain et al., 2004 [27]
*Citrus aurantium*	*p.o.*	anxiolyticlike activity	serotonergic system (5-HT1_A_ receptors)	Costa et al., 2013 [28]
*Citrus bergamia*		decreased stress-induced anxiety	tuning synaptic plasticity	Bagetta et al., 2010 [29]
*Citrus sinensis*	*INH*	acute anxiolytic activity	N/A	Faturi et al., 2010 [30]
*Coriander*	*INH*	increased anxiolytic–antidepressant-like behaviors, and	N/A	Cioanca et al., 2014 [31]
*Cymbopogon citratus*	*p.o.*	anxiolytic-like activity	potentiated GABA_A_ receptor complex	Costa et al., 2011 [13]
*Cymbopogon winterianus* Jowitt; and *C. citratus* (DC) Stapf.	*i.p.*	anticonvulsant activities	via GABAergic neurotransmission	Silva et al., 2010 [32]
*Dysphania graveolens*	*p.o.*	antinociceptive effects	N/A	Déciga-Campos et al., 2017 [33]
*Hyptis mutabilis* (Rich.) *Briq.*	*p.o.*	sedative and central anesthetic activities	no involvement of the GABA_A_-BDZ system	Silva et al., 2013 [34]
*Lavandula angustifolia*	*INH*	anxiolytic-like effects	serotonergic system	Chioca et al., 2013 [35]
*Lippia alba* (Mill.) N.E. Brown	*p.o.*	central anesthetic effect	involvement of the GABAergic system	Heldwein et al., 2012 [36]
*Lemon oil*		anxiolytic, antidepressant-like effects	suppression of DA activity related to enhanced 5-HTnergic neurons	Komiya et al., 2006 [37]
*Melissa officinalis*	*p.o.*	anti-agitation effects in patients and the depressant effects in in-vitro	inhibited GABA-induced currents	Abuhamdah et al., 2008 [38]
*Nigella sativa* Seed lmain components	*p.o.*	potentiation of valproate-induced anticonvulsant effect	increased in GABAergic response	Raza et al., 2008 [39]
Perfume and phytoncid	*in vitro*	anxiolytic anticonvulsant and sedative activity	potentiating GABA_A_ receptors	Aoshima and Hamamoto, 1999 [40]
*Piper guineense*	*INH*	sedative and anxiolytic-like effects	N/A	Tankam and Tto, 2013 [2]
*Pistacia integerrima* Stewart ex Brandis Galls	*in vitro*	relaxant and spasmolytic effects	involvement of β-adrenoceptors and calcium channels	Shirole et al., 2015 [41]
*Salvia sclarea*	*i.p.* or *INH*	anti-depressant-like effect	modulating DAnergic pathway	Seol et al., 2010 [42]
*Syzygium aromaticum*		local anesthesia	Inhibited sodium channels	Huang et al., 2012 [43]
*Tagetes minuta* L	*sc*	anxiogenic-like effects	negative modulation on the GABAergic function	Marin et al., 1998 [44]
*Thymus capitatus* Hoff. et Link.	*p.o.*	antinociceptive activity	via peripheral nervous excitability blockade	Gonçalves et al., 2017 [45]
*Valerian officinalis* L	*p.o.*	sedatives	N/A	Houghton, 1999 [46]

Note: N/A: not applicable; *p.o*.: by mouth, oral; *sc*: subcutaneous injection; *i.p.*: intraperitoneal injection; *INH*: inhalation.

**Table 2:** The summary of pharmacological properties of constituents from essential oils of different plants targeting Na^+^ channels and GABAergic system.

Constituents	Pharmacological Effects	Mechanism of Actions	Authors/Year/Ref.
1,8-Cineole	antinociceptive, smooth muscle relaxant	reduction of excitability of peripheral neurons by blocking voltage-dependent Na^+^ current	Ferreira-da-Silva et al., 2015 [64]
	neuronal excitant	hyperexcitability and epileptiform activity in snail neurons by inhibiting potassium channels	Zeraatpisheh and Vatanparast, 2015 [65]
1-Nitro-2-phenylethane	hypnotic, anti-convulsant and anxiolytic effects	N/A	Oyemitan et al., 2013 [66]
	vasorelaxant effects in rat isolated aortic rings	inhibition of contractile events that are clearly independent of Ca^2+^ influx	Arruda-Barbosa et al., 2014 [67]
	vasorelaxant effects	N/A	Interaminense et al., 2013 [68]
(+)-Borneol	alleviated mechanical hyperalgesia in models of chronic inflammatory and neuropathic pain	enhanced GABA_A_R-mediated GABAergic transmission	JIang et al., 2015 [69]
(+)- and (−)-Borneol	analgesia and anesthesia	positive modulation of GABA_A_R	Granger et al., 2005 [14]
(+)-Dehydrofukinone	sedative or anesthetic effects	interacted with GABAergic receptors; a suppressor of neuronal excitability	Garlet et al., 2016 [70]
(*S*)-Limonene,	Anti-stress effect	*via* the GABAergic system	Zhou et al., 2009 [71]
(*R*)-(+)-Limonene	anxiolytic-like effects	N/A	Lima et al., 2013 [72]
(+)-Dehydrofukinone	sedation, anticonvulsant and anesthesia	potentiated GABA_A_ receptors	Garlet et al., 2017 [73]
α-asarone	antiepileptic effect	enhanced tonic GABAergic inhibition	Huang et al., 2013 [54]
	antiepileptic effect	Na^+^ channel blockade and activation of GABA_A_ receptors	Wang ZJ et al., 2014 [4]
	anticonvulsant	blocked Na^+^ channel, potentiated GABA_A_ receptors	Wang ZJ et al., 2014 [4]
α-(−)-Bisabolol	antinociceptive-like effect	decreased peripheral nerve excitability probably by blockade of voltage-gated Na^+^ channels	Wang YW et al., 2015 [74]
α-Pinene	anxiolytic and hypnotic effects	a partial modulator of GABA_A_ receptors and directly binding to the benzodiazepine binding site of GABA_A_ receptor.	Yang et al., 2016 [75]
β-Citronellol	Hypotensive action	antagonized transmembrane Ca^2+^ influx from the extracellular milieu to produce myorelaxant actions.	Vasconcelos et al., 2016 [76]
(*R*)-(−)-carvone and (*S*)-(+)-carvone	antimanic-like effects	blockade of voltage-gated Na^+^ channels; activating TRPV1 and TRPA1 channels	Nogoceke et al., 2016 [77]
Benzyl benzoate	anxiolytic effect	probably through 5-HTnergic and DAnergic pathways	Alves et al., 2016 [63]
Carvacrol	antinematodal action	nicotinic acetylcholine receptors and GABA receptors	Trailović et al., 2015 [78]
	analgesic activity	reduced excitability of peripheral neurons; reduced voltage-dependent Na^+^ current	Joca et al., 2012, 2015 [79,80]
	anxiolytic effects in the plus-maze test	involvement with GABAergic transmission	Melo et al., 2010 [81]
Estragole	anxiolytic and antimicrobial activities	inhibition of neuronal excitability by blocking Na^+^ channels	Silva-Alves et al., 2013 [82]
Eugenol	local analgesic	inhibition of Na^+^ channels	Vatanparast, 2017 [83]
	analgesic	reduced neuronal hyperexcitability by blocking Na^+^ currents	Huang et al., 2012 [43]
		inhibition of action potentials	Moreira-Lobo et al., 2010 [17]
Isopulegol	pentylenetetrazol-induced convulsions	positive modulation of GABA_A_R and antioxidant properties	Silva et al., 2009 [84]
Linalool	antinociceptive effect	blocked excitability by decreasing the voltage-dependent Na^+^ current in dorsal root ganglion neurons	Leal-Cardoso et al., 2010 [85]
Menthol	analgesia	blocked action potentials in frog sciatic nerves unassociated with TRPM8 activation	Kawasaki et al., 2013 [86]
Methyleugenol	anticonvulsant, antinociceptive and anesthetic activities	agonist of GABA_A_ receptors in cultured hippocampal neurons	Ding et al., 2014 [87]
	antinociceptive effect	inhibition of NMDA receptor-mediated hyperalgesia via GABA_A_ receptors	Yano et al., 2006 [88]
	antinociceptive and anesthetic actions	inhibition of Na_v_1.7 channels	Wang ZJ et al., 2015 [5]
Myrtenol and Verbenol	sedative, anxiolytic and anticonvulsive effects	augments phasic and tonic GABAergic inhibition; positive allosteric modulation of GABA_A_ receptors	van Brederode et al., 2016 [16]
Nerolidol	antinociceptive and anti-inflammatory activity	involvement of the GABAergic system and proinflammatory cytokines	Fonsêca et al., 2016 [89]
Terpinen-4-ol	anticonvulsant effects	involvement of the GABAergic system, and decrease Na^+^ current	Nóbrega et al., 2014 [90]
Thujone	muscle spasms and convulsions	GABA receptor antagonist	Mariani et al., 2016 [91]
Thymol	antinociception	nerve conduction inhibition; activated TRPA1 channels; a positive allosteric modulator of human GABA_A_R	Xu et al., 2015 [92] Priestley et al., 2003 [93]
Thymoquinone	anticonvulsant effects	opioid receptor-mediated increase in GABAergic tone	Hosseinzadeh and Parvardeh, 2004 [94]

Note: N/A: not applicable.
